# Supplementary material for: Validating the Well-Being of Older People (WOOP) Instrument in China
Source: Int J Environ Res Public Health. 2022 Dec 24;20(1):277. doi: 10.3390/ijerph20010277 (PMC9819892; doi:10.3390/ijerph20010277)
Supplement: Supplementary file 1 [file ijerph-20-00277-s001.zip › ijerph-2025206-supplementary.pdf]

Supplementary Table S1. Item response distribution by whether missing data was presented

Item		Excellent	Good	Fair	Poor	Bad	Missing	P-value of Chi-square test
Physical Health	without missing, n=353	111	118	68	36	20	0	
	with missing, n=121	35	31	26	14	15	0	0.097
Mental Health	without missing, n=353	173	111	46	13	10	0	
	with missing, n=121	62	33	14	5	5	2	0.212
Social contacts	without missing, n=353	32	108	149	53	11	0	
	with missing, n=121	3	26	49	16	8	19	0
Receive support	Without missing, n=353	61	181	80	25	6	0	
	with missing, n=121	0	13	18	25	1	64	0
Accept and resilience	Without missing, n=353	12	78	174	67	22	0	
	with missing, n=121	1	11	29	20	9	51	0
Feel useful	Without missing, n=353	12	91	177	53	20	0	
	with missing, n=121	2	26	48	29	8	8	0
Independence	Without missing, n=353	21	68	106	115	43	0	
	with missing, n=121	4	17	14	15	10	61	0
Making ends meet	Without missing, n=353	15	50	131	99	58	0	
	with missing, n=121	1	5	32	44	39	0	0
Living situation	Without missing, n=353	50	181	101	18	3	0	
	with missing, n=121	15	69	30	7	0	0	0.671

Supplementary Table S2. Ordered logistic model of the effect of demographic variables on WOOP item response

Demographic variables		Physical health	Mental health	Social contacts	Receive Support	Acceptance and resilience	Feeling useful	Independence	Making ends meet	Living situation
Gender	Female	-----Reference level-----								
	Male	**0.633**	0.821	1.068	0.827	1.215	0.978	0.826	0.932	0.863
Age		-----Reference level-----								
	60-64					---				
	65-74	**2.306***	1.172	1.162	1.353	**1.85**	**2.226***	**1.925**	**1.113**	1.54
	75-79	**2.574***	1.418	1.27	1.718	**2.253**	**2.166**	**2.104**	**1.364**	0.936
	>=80	**2.71**	0.792	1.637	**2.97**	**8.654***	**5.01***	**3.637***	2.293	2
Marriage		-----Reference level-----								
	Married					--				
	Single	2.379	0.802	0.517	0.461	**0.249**	0.493	0.476	0.38	0.845
	Widowed	1.625	**2.034**	**1.79**	0.627	**1.984**	1.466	1.687	**2.154***	1.003
Living situation		-----Reference level-----								
	Living with partner					-				
	Living with offspring	1.504	0.928	1.452	1.344	**1.99**	1.702	**2.593***	**1.739**	**1.932**
	living alone	0.485	0.599	0.869	1.112	**0.42**	0.585	0.976	**0.442**	1.225
Residence area		-----Reference level-----								
	Township					-				
	Village	0.829	1.314	**1.704**	0.828	**1.597**	**1.529**	1.414	**1.179**	**1.443**
Health condition		-----Reference level-----								
	No health condition					-				
	with at least one health condition	**44.027***	**6.149***	**4.212***	**1.628**	**4.122***	**3.051***	**3.6***	**4.163**	**1.864***

* Suggests significant at 0.01 level, bold font suggests significant at 0.05 level
